# Efficacy of intraperitoneal positive pressure gas expulsion in laparoscopic transabdominal preperitoneal hernioplasty: a retrospective cohort study

**DOI:** 10.1186/s12893-025-02965-y

**Published:** 2025-05-27

**Authors:** Wanpeng He, Bo Chen

**Affiliations:** Department of Gastrointestinal Surgery, The Second People’s Hospital of Neijiang, Neijiang, 641000 China

**Keywords:** Laparoscopic hernioplasty, Preperitoneal space, Mesh fixation, Pneumoperitoneum pressure, Postoperative complications

## Abstract

**Objective:**

To investigate the effects of the pneumoperitoneum positive-pressure exhaust technique on mesh fixation and postoperative recovery in laparoscopic transabdominal preperitoneal prosthetic (TAPP) hernia repair.

**Methods:**

A retrospective cohort analysis was conducted on 655 patients who underwent TAPP between January 2019 and December 2023. Patients were divided into a direct suture group (*n*=304) and a positive-pressure exhaust group (*n*=351) on the basis of preperitoneal space management. In the exhaust group, a 20G needle or drainage tube was placed percutaneously before peritoneal closure. After suturing, 12 mmHg pneumoperitoneum pressure was maintained to evacuate residual gas from the preperitoneal space through the externalized needle/tube. The primary outcomes included postoperative complications (bleeding, mesh infection, seroma, reoperation) and hospitalization duration.

**Results:**

Baseline characteristics were not significantly different (*P*>0.05). Although not statistically significant, there were clinically meaningful differences between the groups; the exhaust group had lower seroma (11.97% vs. 16.78%, *P*=0.079) and mesh infection (0.28% vs. 1.32%, *P*=0.189) incidence rates than the direct suture group did. The exhaust group had a significantly shorter hospital stay than the direct suture group (median 7 vs. 7 days, *P*=0.013) and had a 0% recurrence rate at the 1-year follow-up (vs. 1.32% for the direct suture group).

**Conclusion:**

The positive-pressure exhaust technique facilitates mesh fixation by eliminating dead space through improved tissue apposition. This simple, cost-effective approach may reduce the risk of recurrence, although larger prospective studies are needed to validate its long-term efficacy.

## Introduction

Laparoscopic transabdominal preperitoneal prosthetic (TAPP) repair has become the predominant approach for inguinal hernias because of its minimally invasive nature and rapid recovery benefits. However, postoperative recurrence rates remain at 0.4%−8.5% [1]. Studies indicate that mesh displacement and folding are key contributors to surgical failure [2, 3]. Conventional fixation methods—including tacks, sutures, and biological adhesives—present significant limitations: mechanical fixation may lead to chronic pain or intestinal perforation [4, 5]; suturing prolongs the operative time and carries risks of neural injury [6–8]; and, despite their safety profile, biological adhesives incur high costs [6].

Although mesh fixation-free techniques are well established for totally extraperitoneal (TEP) repair [9–11], their application in TAPP remains controversial. This stems primarily from the dual-cavity structure formed by the abdominal and preperitoneal spaces, which predisposes patients to gas retention, compromising mesh-wall apposition and increasing displacement risk [12].

To address this challenge, we propose a novel technique: intraoperative maintenance of 12 mmHg pneumoperitoneum pressure to actively expel residual gas from the preperitoneal space, achieving three-dimensional fixation through friction among the peritoneum, mesh, and abdominal wall. This approach potentially offers three advantages:

1. Real-time visualization of mesh positioning to prevent folding/displacement
2. Elimination of additional consumables, reducing costs
3. Avoidance of foreign body-related complications

As long-term outcomes of comparable techniques remain unreported, we conducted this large-scale retrospective study (*n*=655) to evaluate the efficacy of positive-pressure gas expulsion on mesh fixation and postoperative recovery, providing new evidence for preperitoneal space management in TAPP procedures.

## Materials and methods

### Data collection

The clinical records of patients who underwent laparoscopic transabdominal preperitoneal prosthetic (TAPP) repair from January 2019 to December 2023 were retrospectively reviewed.

#### Inclusion criteria

1. Unilateral/bilateral inguinal hernia confirmed by physical examination, ultrasonography, or other imaging, scheduled for elective TAPP
2. Age >18 years
3. Complete medical documentation

#### Exclusion criteria

1. Concurrent groin pathologies requiring treatment during TAPP (e.g., varicocele, hydrocele, cryptorchidism)
2. Recurrent hernias undergoing TAPP repair
3. Patients converted to open surgery
4. Patients initially scheduled for TEP repair but switched to TAPP
5. Combined procedures involving other anatomical sites or organs

### Surgical protocol

#### Preoperative evaluation

All patients underwent diagnostic imaging and physical examination. Those with comorbidities (diabetes, COPD, chronic hepatitis, etc.) received specialist evaluations. Patients with uncontrolled baseline conditions or acute exacerbations underwent optimization prior to final anaesthetic assessment.

#### Surgical technique

Standard laparoscopic dissection of the myopectineal orifice was performed. Mesh size selection followed the EHS classification:

- Type I-II defects: 8.5×13.7 cm
- Type III defects: 15×10 cm
  (Bard 3DMax Mesh, Davol Inc.; NMPA registration no. 20173461504)

##### Key steps

- For internal ring defects ≥2 cm: Continuous 2-0 barbed suture (Stratafix, Ethicon) closure; if tension was excessive, the ring was reduced to <3 cm in diameter.
- Direct suture group: Peritoneal closure with 3-0 barbed suture, CO₂ evacuation after fascial closure.
- Positive-pressure exhaust group:
  1. Presuture: A 20G needle was inserted at the lateral 1/3 of the line between the ASIS and the umbilicus (avoiding inferior epigastric vessels) without penetrating the mesh.
  2. Postsuture: A 12 mmHg pneumoperitoneum was maintained to evacuate residual preperitoneal gas through the needle.
  3. The mesh position was verified laparoscopically before needle removal, with final aspiration.
- Drain placement (F12 tube):
  - ◦ Positioned in the dependent preperitoneal space
  - ◦ Opened postperitoneal closure under laparoscopic visualization to confirm mesh apposition
  - ◦ Connected to suction for 48–72 hours

Drain Indications:

1. Difficult peritoneal dissection with significant bleeding/exudate
2. Bilateral TAPP or large operative field
3. Coagulopathy (e.g., cirrhosis, dialysis) or ascites
4. High infection risk (diabetes, HIV, COPD)

#### Follow-up protocol

- In-person visits: 1 week, 1 month, and 2 months postop
- Telephone follow-up at 1 year
- Evaluations included physical examination, pain assessment, and functional recovery
- Ultrasound/CT for symptomatic patients (pain, palpable masses)

#### Statistical analysis

The data were analysed using SPSS 22.0.

- Continuous variables: median (IQR) [M(P25, P75)]; Mann‒Whitney U test
- Categorical variables: frequency [n(%)]; χ^2^ test or Fisher’s exact test
- Significance threshold: *P*<0.05

## Results

### General data

No significant differences were observed in age, sex, or preoperative comorbidities between the two groups (*p*>0.05), as shown in Table 1.

**Table 1:** Comparison of baseline characteristics between groups

**Characteristics**	**Direct suture group (*n*=304)**	**Positive pressure group (*n*=351)**	**Statistical value**	***P* value**
**Sex [n (%)]**			χ^2^=0.254	0.614
Male	298 (98.03)	342 (97.44)		
Female	6 (1.97)	9 (2.56)		
**Age [years, median (IQR)]**	63 (61–68)	66 (60–69)	Z=−1.515	0.130
**Comorbidities [n (%)]**				
Diabetes mellitus	21 (6.91)	30 (8.55)	χ^2^=0.610	0.435
COPD	23 (7.57)	27 (7.69)	χ^2^=0.004	0.952
Liver cirrhosis	9 (2.96)	17 (4.84)	χ^2^=1.515	0.218
Coronary heart disease	23 (7.57)	20 (5.70)	χ^2^=0.927	0.336
Autoimmune disease	7 (2.30)	11 (3.13)	χ^2^=0.421	0.516
Chronic kidney disease	16 (5.26)	15 (4.27)	χ^2^=0.354	0.552

Data are presented as “n (%)” for categorical variables; continuous data are presented as medians (IQRs) because of their nonnormal distribution (Shapiro‒Wilk test, *P*<0.05). Group comparisons were performed using χ^2^ tests for categorical data and the Mann‒Whitney U test (reported as Z values) for nonnormally distributed continuous variables

*COPD* Chronic obstructive pulmonary disease

### Intraoperative data

This table compares the intraoperative variables between the direct suture group and the positive pressure gas expulsion group. The positive pressure group presented significantly higher internal ring closure rates (64.10% vs. 53.29%, *P*=0.005), whereas other parameters, including operative time, blood loss, and drain placement, were comparable between the groups (all *P*>0.05), as shown in Table 2.

**Table 2:** Intraoperative outcomes comparison between groups

**Variables**	**Direct suture group (*n*=304)**	**Positive pressure group (*n*=351)**	**Statistical value**	***P* value**
**Surgical side [n (%)]**			χ^2^=0.237	0.626
Unilateral	268 (88.16)	305 (86.89)		
Bilateral	36 (11.84)	46 (13.11)		
**Internal ring closure [n (%)]**			χ^2^=7.897	0.005
Yes	162 (53.29)	225 (64.10)		
No	142 (46.71)	126 (35.90)		
**Drain placement [n (%)]**			χ^2^=0.005	0.946
Yes	116 (38.16)	133 (37.89)		
No	188 (61.84)	218 (62.11)		
**Operative time [min, median (IQR)]**	70 (62–78)	70 (66–78)	Z=−0.681	0.496
**Blood loss [ml, median (IQR)]**	5 (5–15)	5 (5–15)	Z=−0.495	0.620

Data are presented as “n (%)” for categorical variables; continuous data are presented as medians (IQRs) because of their nonnormal distribution (Shapiro‒Wilk *P*<.05). Group comparisons were performed using χ^2^ tests for proportions and Mann‒Whitney U tests (reported as *Z* values) for nonnormally distributed continuous variables (operative time and blood loss)

### Postoperative data

The positive pressure group had no patients who required reoperation. In the direct suture group, 8 patients experienced postoperative bleeding (4 with preperitoneal haemorrhage, 2 with umbilical port site bleeding, and 2 with lateral abdominal wall port bleeding). Among these patients, 2 developed preperitoneal haematomas requiring laparoscopic evacuation (1 from spermatic vessel bleeding and 1 from diffuse oozing without an identifiable source), with 1 patient developing recurrent hernia at 7 months postoperatively.

The positive pressure group included 5 cases of bleeding (1 preperitoneal, 2 umbilical ports, and 1 lateral port), all of which were managed successfully with drainage and haemostatic therapy. In the direct suture group, 4 patients developed mesh infection; 2 patients required mesh removal due to failed antibiotic/drainage therapy, and both of them experienced hernia recurrence at 3 and 5 months postoperatively. The positive pressure group had only 1 mesh infection case that resolved with conservative treatment.

Recurrence within 1 year occurred in 4 direct suture cases (1.32%) but not in the positive pressure group (*P*=0.054, Fisher’s exact test). Type III-IV seromas developed in 51 direct suture cases (16.78%), with 14 requiring percutaneous drainage, compared with 42 cases (11.97%) in the positive pressure group (*P*=0.079). No significant differences were observed in overall complication rates or reintervention needs (*P*>0.05), but the positive pressure group had significantly shorter hospitalizations (median 7 [IQR 6–9] vs. 7 [5–10] days, *P*=0.013), as shown in Table 3.

**Table 3:** Comparison of postoperative recovery outcomes between groups

**Outcomes**	**Direct suture group (*n*=304)**	**Positive pressure group (*n*=351)**	**Statistical value**	***P* value**
**Postoperative complications [n (%)]**				
Bleeding	8 (2.63)	5 (1.42)	χ^2^=1.220	0.269
Mesh infection	4 (1.32)	1 (0.28)	Fisher’s exact	0.189
Seroma (type III-IV)	51 (16.78)	42 (11.97)	χ^2^=3.094	0.079
Hernia recurrence	4 (1.32)	0 (0)	Fisher’s exact	0.054
Reoperation	4 (1.32)	0 (0)	Fisher’s exact	0.054
**Postoperative drainage [n (%)]**	14 (5.92)	15 (4.27)	χ^2^=0.924	0.336
**Hospital stay [days, median (IQR)]**	7 (5–10)	7 (6–9)	Z=−2.492	0.013

Data are presented as “n (%)” for categorical variables; continuous data are presented as medians (IQRs) because of their nonnormal distribution (Shapiro‒Wilk *P*<.05). Group comparisons were performed using χ^2^ tests or Fisher’s exact test (for outcomes with expected cell counts <5) for categorical data and the Mann‒Whitney U test (reported as the Z value) for continuous variables. A type III-IV seroma is a clinically significant seroma requiring intervention

## Discussion

### Key determinants of TAPP surgical outcomes

The success of TAPP primarily hinges on two critical factors: (1) complete anatomical coverage of the myopectineal orifice by the mesh and (2) rapid adhesion formation at the mesh‒tissue interface. In the present study, we identified preperitoneal space stability as the pivotal element governing these outcomes. Given the limited mechanical strength of peritoneal tissue (average thickness: 0.1–0.3 mm), postoperative bleeding or fluid accumulation may increase intracavitary pressure, causing peritoneal tenting and subsequent mesh deformation/migration. More significantly, residual CO₂-induced “gas pocket effects” substantially delay tissue adhesion, creating a window for early mesh displacement [13–15].

### Evolution of fixation-free techniques

Although some studies suggest that nonfixation mesh placement in TAPP does not increase recurrence or complications [9, 16, 17], most studies lack detailed technical descriptions regarding the management of the dead space between the mesh, peritoneum, and abdominal wall. This knowledge gap has hindered widespread adoption. Claus et al. [18] demonstrated through radiographically marked meshes that early post-TEP mobilization (including leg raising) does not cause mesh displacement, thereby reducing postoperative pain and bleeding risks—a conclusion corroborated by Yilmaz [19]. This stability likely stems from the inherent negative-pressure environment of TEP, which eliminates dead space and generates bidirectional frictional forces. Soeta’s technique [20] of preperitoneal negative-pressure aspiration, which provides real-time visual confirmation of mesh apposition, inspired our methodology.

### Mechanistic advantages of positive-pressure exhaust technique

Our innovation creates two isolated compartments (mesh space vs. the abdominal cavity) and utilizes 12 mmHg pneumoperitoneum to actively evacuate preperitoneal gas via needles/drains, resulting in the following:

1. Triple fixation mechanism(under laparoscopic visualization):
  ◦ Negative-pressure adhesion at the peritoneum‒mesh interface (via drainage)
  ◦ Frictional forces between the mesh and the abdominal wall [21]
  ◦ Elimination of potential displacement spaces
2. Higher internal ring closure rate (64.10% vs. 53.29%, *P*= 0.005), reducing dead space and enhancing mesh support.

### Clinical outcomes and comparative analysis

In our series of 655 TAPP cases, the positive-pressure group showed clinically meaningful (albeit statistically nonsignificant) improvements:

- Seroma (11.97% vs. 16.78%, *P*= 0.079)
- Mesh infection (0.28% vs. 1.32%, *P* = 0.189)

Notably, the zero recurrence rate at the 1-year follow-up (vs. 1.32% in controls) outperformed Mayer’s reported rate of 1.1% [17]. The incidence of seroma in the control group (16.78%) slightly exceeded that reported by Saini (14.1%) [22], whereas the study group (11.97%) demonstrated marked improvement. A statistically significant reduction in hospitalization duration (median 7 vs. 7 days, *P*=0.013) reflected technical refinements.

### Re-evaluation of the drainage strategy

Contrary to the literature neglect, our 35% drainage utilization proved critical:

- Managed 2 preperitoneal haemorrhages and 2 mesh infections in controls
- Resolved 1 haemorrhage and 1 infection in the study group
- Undrained cases exhibited delayed complication recognition

#### Supporting evidence

Fang/Luo [23, 24] reported reduced seroma rates and enhanced scrotal hernia recovery, whereas Fan/Prassas [25, 26] confirmed no increased infection risk with short-term drainage (<48 h). Negative-pressure systems may further promote early mesh incorporation.

### Technical comparisons and safety

Traditional transabdominal suction methods carry risks:

- Peritoneal laceration
- Mesh exposure
- Internal herniation through peritoneal defects [5, 15–17]
- Uncontrolled suction-induced microvascular injury

Our pressure-regulated exhaust technique avoids these pitfalls while matching Soeta’s aspiration [20] in space reduction. The higher internal ring closure rate—a previously underappreciated factor—minimizes residual sac pressure effects on the preperitoneal space.

## Conclusions and limitations

This technique offers three advantages:

- Real-time mesh positioning assessment
- Multimodal fixation without additional devices
- Simplified dead space management

The study limitations include the following:

1. Potential selection bias inherent to retrospective design
2. Intermediate-term (1-year) recurrence follow-up
3. Exclusive use of Bard 3DMax heavyweight mesh—lightweight mesh applicability requires further study
4. Potential bias from high internal ring suture rates and frequent drain use in this cohort

## Data Availability

The datasets used and/or analyzed during the current study are available from the corresponding author on reasonable request.

## Abbreviations

TAPP: Transabdominal Preperitoneal Prosthetic
TEP: Totally Extraperitoneal Prosthetic
COPD: Chronic Obstructive Pulmonary Disease
